# 2-Methyl-3-nitro-*N*-{(*E*)-[5-(4-nitro­phen­yl)furan-2-yl]methyl­idene}aniline

**DOI:** 10.1107/S1600536812033818

**Published:** 2012-08-04

**Authors:** Merve Pekdemir, Şamil Işık, Sümeyye Gümüş, Erbil Ağar, Sema Öztürk Yıldırım, Ray J. Butcher

**Affiliations:** a Department of Physics, Ondokuz Mayıs University, TR-55139 Samsun, Turkey; b Department of Physics, Faculty of Arts and Sciences, Ondokuz Mayıs University, Kurupelit, TR-55139 Samsun, Turkey; c Department of Chemistry, Faculty of Arts and Sciences, Ondokuz Mayıs University, Kurupelit, TR-55139 Samsun, Turkey; d Faculty of Sciences, Department of Physics, Erciyes University, 38039 Kayseri, Turkey; e Howard University, College of Arts & Sciences, Department of Chemistry, 525 College Street NW, Washington, DC 20059, USA

## Abstract

In the title Schiff-base type compound, C_18_H_13_N_3_O_5_, the central furan ring makes dihedral angles of 12.80 (7) and 51.43 (4)° with the terminal benzene rings. The dihedral angle between the benzene rings is 45.43 (3)°. In the crystal, C—H⋯O hydrogen bonds link the mol­ecules into layers parallel to (010). In addition, there are π–π stacking inter­actions within the layer [centroid–centroid distance = 3.584 (1) Å] and between the layers [centroid–centroid distance 3.751 (1) Å].

## Related literature

For similar Schiff bases, see: Yamada *et al.* (2002 ▶); Cukurovali *et al.* (2002 ▶); Isloor *et al.* (2009 ▶); Abu Thaher *et al.* (2012 ▶). For the biological activity of Schiff bases, see: Vijesh *et al.* (2010 ▶); Tarafder *et al.* (2002 ▶); Ghorab *et al.* (2010 ▶); Ali *et al.* (2002 ▶). For standard bond lengths, see: Allen *et al.* (1987 ▶).
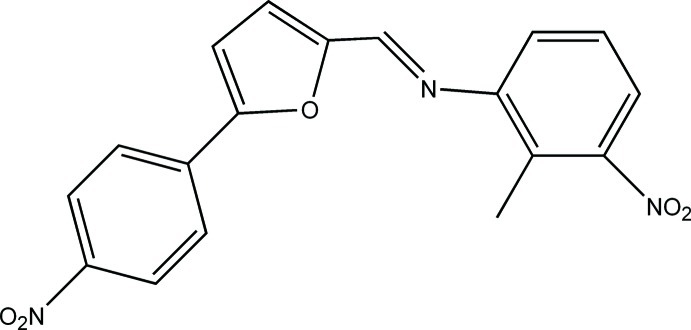


## Experimental

### 

#### Crystal data

C_18_H_13_N_3_O_5_
*M* *_r_* = 351.31
Monoclinic, 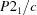

*a* = 10.9026 (3) Å
*b* = 10.2798 (3) Å
*c* = 14.2962 (3) Å
β = 101.529 (2)°
*V* = 1569.94 (7) Å^3^
*Z* = 4
Cu *K*α radiation
μ = 0.93 mm^−1^
*T* = 123 K
0.50 × 0.40 × 0.40 mm

#### Data collection

Oxford Diffraction Gemini-R diffractometer
Absorption correction: multi-scan [*CrysAlis RED* (Oxford Diffraction, 2007 ▶), and Clark & Reid (1995 ▶)] *T*_min_ = 0.671, *T*_max_ = 0.688
6460 measured reflections
3171 independent reflections
2764 reflections with *I* > 2σ(*I*)
*R*_int_ = 0.028

#### Refinement

*R*[*F* ^2^ > 2σ(*F* ^2^)] = 0.042
*wR*(*F* ^2^) = 0.118
*S* = 1.05
3171 reflections
236 parameters
H-atom parameters constrained
Δρ_max_ = 0.31 e Å^−3^
Δρ_min_ = −0.23 e Å^−3^

### 

Data collection: *CrysAlis CCD* (Oxford Diffraction, 2007 ▶); cell refinement: *CrysAlis CCD*; data reduction: *CrysAlis RED* (Oxford Diffraction, 2007 ▶); program(s) used to solve structure: *SHELXS97* (Sheldrick, 2008 ▶); program(s) used to refine structure: *SHELXL97* (Sheldrick, 2008 ▶); molecular graphics: *SHELXTL* (Sheldrick, 2008 ▶); software used to prepare material for publication: *SHELXTL*.

## Supplementary Material

Crystal structure: contains datablock(s) I, global. DOI: 10.1107/S1600536812033818/gk2492sup1.cif

Structure factors: contains datablock(s) I. DOI: 10.1107/S1600536812033818/gk2492Isup2.hkl

Supplementary material file. DOI: 10.1107/S1600536812033818/gk2492Isup3.cml

Additional supplementary materials:  crystallographic information; 3D view; checkCIF report

## Figures and Tables

**Table 1:** Hydrogen-bond geometry (Å, °)

*D*—H⋯*A*	*D*—H	H⋯*A*	*D*⋯*A*	*D*—H⋯*A*
C3—H3*A*⋯O5^i^	0.93	2.58	3.4895 (19)	165
C13—H13*A*⋯O1^ii^	0.93	2.53	3.432 (2)	162
C14—H14*A*⋯O2^iii^	0.93	2.55	3.361 (2)	147

Symmetry codes: (i) 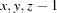; (ii) 
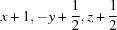
; (iii) 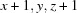.

**Fig. 1.**
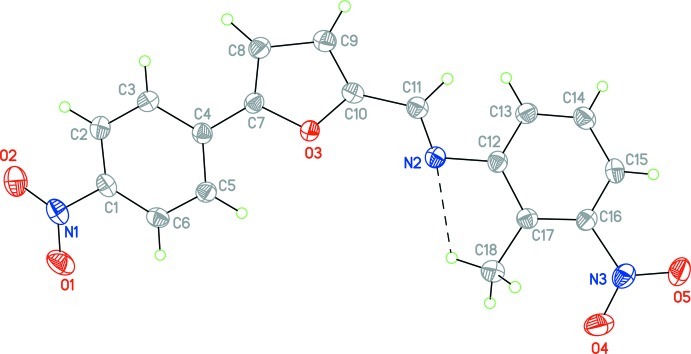
View of the molecular structure of the title compound with the atom-numbering scheme. Displacement ellipsoids are drawn at the 50% probability level for non-hydrogen atoms. The intramolecular interaction is shown as a dashed line.

**Fig. 2.**
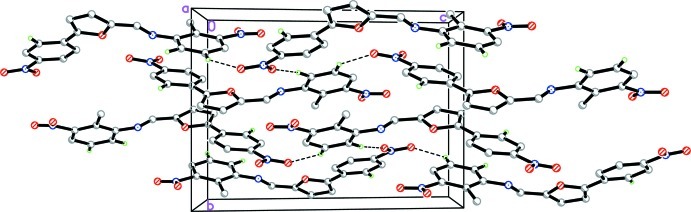
The crystal structure of the title compound, viewed along the a axis. H atoms not involved in hydrogen bonds (dashed lines) have been omitted for clarity.

## supplementary crystallographic information

### Comment

Schiff bases have been very important as ligands in the area of coordination chemistry. These ligands and their metal complexes have shown important activity in the field of biology in the past years. Several new examples have been tested for their antitumor, antimicrobial, anticancer and antibacterial activities (Ali *et al.*, 2002; Ghorab *et al.*, 2010; Tarafder *et al.*, 2002; Vijesh *et al.*, 2010). In view of these facts, the aim of this present study is to obtain a structure of the Schiff base, 2-methyl-3-nitro-*N*-{(*E*)-[5-(4-nitrophenyl)furan-2-yl]methylideneaniline.

In the title compound (Fig 1), the molecule presents an E configuration with the 2-(4-nitrophenyl)furan group opposite to 1-methyl-2-nitrobenzene group about the N2 ═C11 double bond. This N2 ═C11 double bond distance [1.2783 (19) Å] is longer than the N ═C typical bond distance (Allen *et al.*, 1987), probably due to π conjugation along all the molecule. The torsion angle is 171.79 (14) ° formed by C10—C11—N2—C12. All other bond lengths and angles are within normal ranges (Allen *et al.*, 1987). The central furan ring (C7—C10/O3) is planar, with an r.m.s. deviation for fitted atoms of 0.0019 Å. This plane makes dihedral angles of 12.80 (7) and 51.43 (4) ° with the terminal benzene rings C1—C6 and C12—C17, respectively. The dihedral angle between the two benzene ring is 45.43 (3)°.

In the crystal, C—H···O intermolecular interactions are observed (Table 1) as well as π–π stacking interactions [*Cg*1···*Cg*3 (-*x*,1 - *y*,-*z*) = 3.584 (1) Å and *Cg*3···*Cg*3 (*x*, 1 + *y*, *z*) = 3.751 (1) Å, where *Cg*1(O3/C7—C10) and *Cg*3(C12—C17) are the centroids of the furan and benzene ring, respectively] (Fig. 2).

### Experimental

The title compound was prepared by refluxing a mixture containing 5-(4-nitrophenyl)furan-2 carbaldehyde (0.011 g 0.051 mmol) and 2-methyl-3-nitroaniline (0.0077 g 0.051 mmol) in 40 ml of ethanol. The reaction mixture was stirred for 1 h under reflux. The crystals suitable for X-ray analysis were obtained from ethanol by slow evaporation (yield 62%; m.p: 471–474 K).

### Refinement

All H atoms were positioned geometrically and refined using a riding model with C–H = 0.93–0.96 Å and *U*_iso_(H) = 1.2 or 1.5 *U*_eq_(C). A rotating-group model was applied for the methyl groups.

### Crystal data

**Table:**

C_18_H_13_N_3_O_5_	*F*(000) = 728
*M**_r_* = 351.31	*D*_x_ = 1.486 Mg m^−^^3^
Monoclinic, *P*2_1_/*c*	Cu *K*α radiation, λ = 1.5418 Å
Hall symbol: -P 2ybc	Cell parameters from 3039 reflections
*a* = 10.9026 (3) Å	θ = 3.2–75.5°
*b* = 10.2798 (3) Å	µ = 0.93 mm^−^^1^
*c* = 14.2962 (3) Å	*T* = 123 K
β = 101.529 (2)°	Block, light yellow
*V* = 1569.94 (7) Å^3^	0.50 × 0.40 × 0.40 mm
*Z* = 4	

### Data collection

**Table:**

Oxford Diffraction Gemini-R diffractometer	3171 independent reflections
Radiation source: Enhance (Cu) X-ray Source	2764 reflections with *I* > 2σ(*I*)
Graphite monochromator	*R*_int_ = 0.028
Detector resolution: 10.5081 pixels mm^-1^	θ_max_ = 75.7°, θ_min_ = 4.1°
ω scans	*h* = −12→13
Absorption correction: multi-scan [*CrysAlis RED* (Oxford Diffraction, 2007), and Clark & Reid (1995)]	*k* = −12→12
*T*_min_ = 0.671, *T*_max_ = 0.688	*l* = −17→7
6460 measured reflections	

### Refinement

**Table:**

Refinement on *F*^2^	Primary atom site location: structure-invariant direct methods
Least-squares matrix: full	Secondary atom site location: difference Fourier map
*R*[*F*^2^ > 2σ(*F*^2^)] = 0.042	Hydrogen site location: inferred from neighbouring sites
*wR*(*F*^2^) = 0.118	H-atom parameters constrained
*S* = 1.05	*w* = 1/[σ^2^(*F*_o_^2^) + (0.0671*P*)^2^ + 0.3233*P*] where *P* = (*F*_o_^2^ + 2*F*_c_^2^)/3
3171 reflections	(Δ/σ)_max_ < 0.001
236 parameters	Δρ_max_ = 0.31 e Å^−^^3^
0 restraints	Δρ_min_ = −0.23 e Å^−^^3^

### Special details

**Table:**

Geometry. All e.s.d.'s (except the e.s.d. in the dihedral angle between two l.s. planes) are estimated using the full covariance matrix. The cell e.s.d.'s are taken into account individually in the estimation of e.s.d.'s in distances, angles and torsion angles; correlations between e.s.d.'s in cell parameters are only used when they are defined by crystal symmetry. An approximate (isotropic) treatment of cell e.s.d.'s is used for estimating e.s.d.'s involving l.s. planes.
Refinement. Refinement of *F*^2^ against ALL reflections. The weighted *R*-factor *wR* and goodness of fit *S* are based on *F*^2^, conventional *R*-factors *R* are based on *F*, with *F* set to zero for negative *F*^2^. The threshold expression of *F*^2^ > σ(*F*^2^) is used only for calculating *R*-factors(gt) *etc*. and is not relevant to the choice of reflections for refinement. *R*-factors based on *F*^2^ are statistically about twice as large as those based on *F*, and *R*- factors based on ALL data will be even larger.

### Fractional atomic coordinates and isotropic or equivalent isotropic displacement parameters (Å^2^)

**Table:**

	*x*	*y*	*z*	*U*_iso_*/*U*_eq_	
N1	−0.22774 (12)	0.22828 (14)	−0.24867 (10)	0.0309 (3)	
N2	0.28795 (11)	0.40005 (12)	0.30426 (9)	0.0224 (3)	
N3	0.26983 (13)	0.41136 (13)	0.64618 (9)	0.0281 (3)	
O1	−0.32515 (11)	0.21084 (14)	−0.21964 (10)	0.0412 (3)	
O2	−0.21821 (12)	0.20872 (15)	−0.33171 (9)	0.0448 (3)	
O3	0.20668 (9)	0.40126 (10)	0.10398 (7)	0.0206 (2)	
O4	0.15778 (13)	0.43153 (18)	0.63095 (9)	0.0537 (4)	
O5	0.33489 (12)	0.41218 (14)	0.72703 (8)	0.0403 (3)	
C1	−0.11759 (13)	0.27566 (15)	−0.18146 (10)	0.0247 (3)	
C2	−0.01288 (15)	0.31134 (16)	−0.21649 (11)	0.0280 (3)	
H2A	−0.0121	0.3041	−0.2812	0.034*	
C3	0.09029 (14)	0.35784 (16)	−0.15327 (10)	0.0258 (3)	
H3A	0.1611	0.3832	−0.1756	0.031*	
C4	0.08916 (13)	0.36719 (14)	−0.05598 (10)	0.0209 (3)	
C5	−0.01791 (13)	0.32932 (14)	−0.02242 (10)	0.0224 (3)	
H5A	−0.0187	0.3345	0.0424	0.027*	
C6	−0.12221 (13)	0.28430 (14)	−0.08556 (11)	0.0242 (3)	
H6A	−0.1940	0.2603	−0.0641	0.029*	
C7	0.20003 (13)	0.41645 (14)	0.00792 (10)	0.0204 (3)	
C8	0.30432 (14)	0.47801 (15)	−0.00873 (10)	0.0236 (3)	
H8A	0.3217	0.4999	−0.0679	0.028*	
C9	0.38082 (14)	0.50202 (15)	0.08172 (10)	0.0236 (3)	
H9A	0.4585	0.5428	0.0936	0.028*	
C10	0.31886 (13)	0.45401 (14)	0.14813 (10)	0.0215 (3)	
C11	0.35804 (13)	0.44480 (14)	0.25000 (10)	0.0222 (3)	
H11A	0.4382	0.4727	0.2778	0.027*	
C12	0.34377 (13)	0.38033 (14)	0.40136 (10)	0.0216 (3)	
C13	0.46195 (14)	0.32360 (15)	0.42596 (11)	0.0248 (3)	
H13A	0.5056	0.3028	0.3783	0.030*	
C14	0.51519 (14)	0.29778 (15)	0.52044 (11)	0.0270 (3)	
H14A	0.5945	0.2608	0.5361	0.032*	
C15	0.45026 (14)	0.32705 (15)	0.59109 (10)	0.0248 (3)	
H15A	0.4849	0.3099	0.6548	0.030*	
C16	0.33171 (13)	0.38282 (14)	0.56546 (10)	0.0221 (3)	
C17	0.27330 (13)	0.41176 (14)	0.47145 (10)	0.0208 (3)	
C18	0.14791 (14)	0.47584 (16)	0.43966 (11)	0.0267 (3)	
H18A	0.1486	0.5596	0.4694	0.040*	
H18B	0.0841	0.4227	0.4577	0.040*	
H18C	0.1310	0.4861	0.3715	0.040*	

### Atomic displacement parameters (Å^2^)

**Table:**

	*U*^11^	*U*^22^	*U*^33^	*U*^12^	*U*^13^	*U*^23^
N1	0.0263 (7)	0.0291 (7)	0.0333 (7)	0.0015 (5)	−0.0037 (5)	−0.0025 (6)
N2	0.0224 (6)	0.0234 (6)	0.0213 (6)	0.0002 (5)	0.0040 (5)	−0.0015 (5)
N3	0.0339 (7)	0.0280 (7)	0.0237 (6)	−0.0029 (6)	0.0091 (5)	0.0007 (5)
O1	0.0234 (6)	0.0482 (8)	0.0492 (7)	−0.0046 (5)	0.0003 (5)	−0.0067 (6)
O2	0.0407 (7)	0.0580 (9)	0.0306 (6)	−0.0050 (6)	−0.0051 (5)	−0.0087 (6)
O3	0.0211 (5)	0.0228 (5)	0.0177 (5)	−0.0007 (4)	0.0034 (4)	−0.0005 (4)
O4	0.0355 (7)	0.0957 (13)	0.0336 (7)	0.0182 (8)	0.0155 (5)	0.0061 (7)
O5	0.0425 (7)	0.0572 (8)	0.0218 (6)	−0.0081 (6)	0.0076 (5)	−0.0048 (5)
C1	0.0221 (7)	0.0231 (7)	0.0263 (7)	0.0025 (6)	−0.0015 (6)	−0.0012 (6)
C2	0.0282 (7)	0.0338 (8)	0.0212 (7)	0.0010 (6)	0.0031 (6)	−0.0012 (6)
C3	0.0238 (7)	0.0314 (8)	0.0230 (7)	0.0001 (6)	0.0067 (5)	0.0022 (6)
C4	0.0213 (7)	0.0194 (7)	0.0215 (7)	0.0028 (5)	0.0031 (5)	0.0013 (5)
C5	0.0234 (7)	0.0221 (7)	0.0220 (7)	0.0029 (6)	0.0056 (5)	0.0008 (6)
C6	0.0209 (7)	0.0226 (7)	0.0295 (8)	0.0024 (6)	0.0061 (6)	0.0025 (6)
C7	0.0229 (7)	0.0206 (7)	0.0182 (7)	0.0043 (5)	0.0051 (5)	0.0020 (5)
C8	0.0234 (7)	0.0265 (7)	0.0218 (7)	0.0022 (6)	0.0064 (5)	0.0030 (6)
C9	0.0212 (7)	0.0245 (7)	0.0254 (7)	−0.0009 (6)	0.0052 (5)	0.0004 (6)
C10	0.0190 (6)	0.0206 (7)	0.0247 (7)	−0.0002 (5)	0.0043 (5)	−0.0011 (6)
C11	0.0217 (7)	0.0208 (7)	0.0239 (7)	−0.0005 (6)	0.0038 (5)	−0.0022 (6)
C12	0.0229 (7)	0.0211 (7)	0.0205 (7)	−0.0037 (6)	0.0037 (5)	−0.0014 (5)
C13	0.0246 (7)	0.0262 (7)	0.0246 (7)	0.0001 (6)	0.0073 (6)	−0.0020 (6)
C14	0.0226 (7)	0.0270 (8)	0.0299 (8)	0.0025 (6)	0.0016 (6)	−0.0013 (6)
C15	0.0271 (7)	0.0234 (7)	0.0219 (7)	−0.0036 (6)	−0.0001 (5)	0.0000 (6)
C16	0.0254 (7)	0.0205 (7)	0.0215 (7)	−0.0050 (6)	0.0069 (5)	−0.0023 (5)
C17	0.0204 (7)	0.0189 (7)	0.0236 (7)	−0.0033 (5)	0.0053 (5)	−0.0012 (5)
C18	0.0237 (7)	0.0295 (8)	0.0270 (7)	0.0022 (6)	0.0056 (6)	0.0001 (6)

### Geometric parameters (Å, º)

**Table:**

N1—O1	1.2282 (18)	C7—C8	1.363 (2)
N1—O2	1.2288 (19)	C8—C9	1.413 (2)
N1—C1	1.4633 (19)	C8—H8A	0.9300
N2—C11	1.2783 (19)	C9—C10	1.363 (2)
N2—C12	1.4145 (18)	C9—H9A	0.9300
N3—O4	1.2151 (19)	C10—C11	1.437 (2)
N3—O5	1.2294 (18)	C11—H11A	0.9300
N3—C16	1.4779 (18)	C12—C13	1.394 (2)
O3—C7	1.3696 (16)	C12—C17	1.417 (2)
O3—C10	1.3711 (17)	C13—C14	1.385 (2)
C1—C2	1.385 (2)	C13—H13A	0.9300
C1—C6	1.385 (2)	C14—C15	1.378 (2)
C2—C3	1.380 (2)	C14—H14A	0.9300
C2—H2A	0.9300	C15—C16	1.394 (2)
C3—C4	1.397 (2)	C15—H15A	0.9300
C3—H3A	0.9300	C16—C17	1.399 (2)
C4—C5	1.403 (2)	C17—C18	1.504 (2)
C4—C7	1.453 (2)	C18—H18A	0.9600
C5—C6	1.383 (2)	C18—H18B	0.9600
C5—H5A	0.9300	C18—H18C	0.9600
C6—H6A	0.9300		
			
O1—N1—O2	123.25 (14)	C10—C9—H9A	126.6
O1—N1—C1	118.58 (14)	C8—C9—H9A	126.6
O2—N1—C1	118.17 (14)	C9—C10—O3	110.08 (12)
C11—N2—C12	117.06 (12)	C9—C10—C11	129.92 (13)
O4—N3—O5	122.54 (14)	O3—C10—C11	119.85 (12)
O4—N3—C16	119.57 (13)	N2—C11—C10	123.11 (13)
O5—N3—C16	117.89 (13)	N2—C11—H11A	118.4
C7—O3—C10	106.26 (11)	C10—C11—H11A	118.4
C2—C1—C6	122.47 (14)	C13—C12—N2	120.15 (13)
C2—C1—N1	118.59 (14)	C13—C12—C17	121.44 (13)
C6—C1—N1	118.94 (14)	N2—C12—C17	118.28 (13)
C3—C2—C1	118.55 (14)	C14—C13—C12	120.82 (14)
C3—C2—H2A	120.7	C14—C13—H13A	119.6
C1—C2—H2A	120.7	C12—C13—H13A	119.6
C2—C3—C4	120.52 (14)	C15—C14—C13	119.77 (14)
C2—C3—H3A	119.7	C15—C14—H14A	120.1
C4—C3—H3A	119.7	C13—C14—H14A	120.1
C3—C4—C5	119.67 (13)	C14—C15—C16	118.84 (14)
C3—C4—C7	118.58 (13)	C14—C15—H15A	120.6
C5—C4—C7	121.76 (13)	C16—C15—H15A	120.6
C6—C5—C4	120.11 (13)	C15—C16—C17	124.04 (14)
C6—C5—H5A	119.9	C15—C16—N3	114.85 (13)
C4—C5—H5A	119.9	C17—C16—N3	121.10 (13)
C5—C6—C1	118.68 (14)	C16—C17—C12	115.08 (13)
C5—C6—H6A	120.7	C16—C17—C18	126.53 (13)
C1—C6—H6A	120.7	C12—C17—C18	118.35 (13)
C8—C7—O3	110.44 (13)	C17—C18—H18A	109.5
C8—C7—C4	132.08 (13)	C17—C18—H18B	109.5
O3—C7—C4	117.48 (12)	H18A—C18—H18B	109.5
C7—C8—C9	106.36 (13)	C17—C18—H18C	109.5
C7—C8—H8A	126.8	H18A—C18—H18C	109.5
C9—C8—H8A	126.8	H18B—C18—H18C	109.5
C10—C9—C8	106.86 (13)		
			
O1—N1—C1—C2	−171.75 (15)	C7—O3—C10—C9	0.44 (15)
O2—N1—C1—C2	7.8 (2)	C7—O3—C10—C11	−175.51 (13)
O1—N1—C1—C6	7.7 (2)	C12—N2—C11—C10	171.79 (14)
O2—N1—C1—C6	−172.80 (15)	C9—C10—C11—N2	178.09 (15)
C6—C1—C2—C3	−0.3 (2)	O3—C10—C11—N2	−6.9 (2)
N1—C1—C2—C3	179.05 (14)	C11—N2—C12—C13	−43.4 (2)
C1—C2—C3—C4	0.7 (2)	C11—N2—C12—C17	140.62 (14)
C2—C3—C4—C5	−0.2 (2)	N2—C12—C13—C14	−176.78 (14)
C2—C3—C4—C7	179.87 (14)	C17—C12—C13—C14	−0.9 (2)
C3—C4—C5—C6	−0.7 (2)	C12—C13—C14—C15	0.7 (2)
C7—C4—C5—C6	179.25 (13)	C13—C14—C15—C16	−0.2 (2)
C4—C5—C6—C1	1.0 (2)	C14—C15—C16—C17	−0.1 (2)
C2—C1—C6—C5	−0.5 (2)	C14—C15—C16—N3	−179.66 (13)
N1—C1—C6—C5	−179.91 (13)	O4—N3—C16—C15	−163.26 (16)
C10—O3—C7—C8	−0.50 (15)	O5—N3—C16—C15	15.9 (2)
C10—O3—C7—C4	179.55 (12)	O4—N3—C16—C17	17.2 (2)
C3—C4—C7—C8	12.5 (2)	O5—N3—C16—C17	−163.65 (14)
C5—C4—C7—C8	−167.42 (15)	C15—C16—C17—C12	−0.1 (2)
C3—C4—C7—O3	−167.53 (13)	N3—C16—C17—C12	179.45 (12)
C5—C4—C7—O3	12.5 (2)	C15—C16—C17—C18	−177.78 (14)
O3—C7—C8—C9	0.36 (17)	N3—C16—C17—C18	1.8 (2)
C4—C7—C8—C9	−179.70 (15)	C13—C12—C17—C16	0.6 (2)
C7—C8—C9—C10	−0.08 (17)	N2—C12—C17—C16	176.52 (12)
C8—C9—C10—O3	−0.23 (17)	C13—C12—C17—C18	178.50 (14)
C8—C9—C10—C11	175.19 (15)	N2—C12—C17—C18	−5.6 (2)

### Hydrogen-bond geometry (Å, º)

**Table:**

*D*—H···*A*	*D*—H	H···*A*	*D*···*A*	*D*—H···*A*
C18—H18*C*···N2	0.96	2.30	2.8047 (19)	112
C3—H3*A*···O5^i^	0.93	2.58	3.4895 (19)	165
C13—H13*A*···O1^ii^	0.93	2.53	3.432 (2)	162
C14—H14*A*···O2^iii^	0.93	2.55	3.361 (2)	147

Symmetry codes: (i) *x*, *y*, *z*−1; (ii) *x*+1, −*y*+1/2, *z*+1/2; (iii) *x*+1, *y*, *z*+1.
